# Impact of Bulky Substituents on the Singlet Arylnitrene Ring Enlargement

**DOI:** 10.1021/acs.joc.5c00720

**Published:** 2025-07-28

**Authors:** Holger F. Bettinger

**Affiliations:** Institut für Organische Chemie, 9188 Eberhard Karls Universität Tübingen, Auf der Morgenstelle 18, 72076 Tübingen, Germany

## Abstract

Substituents of the hydrindacene type allow the isolation of crystalline stable triplet arylnitrenes from photodecomposition of the corresponding aryl azides, while phenyl azide is known to produce singlet phenylnitrene that reacts to didehydroazepine via benzazirine. Comparison of computed potential energy surfaces for ring enlargement of singlet arylnitrenes suggests that bulky groups of the hydrindacene type should not significantly change the rates of formation of didehydroazepines compared to phenylnitrene. Thus, didehydroazepines could be trappable intermediates en route to stable triplet arylnitrenes.

## Graphical abstract

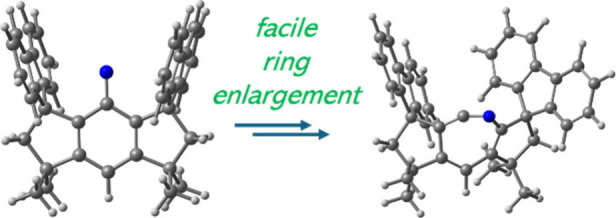

Phenylnitrene (**1a**), generated photochemically from phenyl azide, is known to quickly isomerize via a benzazirine intermediate (**2a**) to didehydroazepine (also called ketenimine, **3a**) that typically undergoes follow-up reactions (Scheme 1).^1^ The reaction of **2** to **3** can proceed by heavy-atom tunneling at 10 K, but due to the small barrier, it is fast at room temperature.^2–4^ The recent reports^5,6^ of kinetically stabilized crystalline triplet arylnitrenes (**1b**, **1c**; Scheme 1) pose the question as to why the photolysis of the corresponding azide precursor in solution at room temperature does not result in singlet arylnitrene rearrangement but rather in formation of the stable triplet in medium to high yields (52–80%). The bulky groups of **1b** and **1c** preclude the typically observed dimerization of triplet arylnitrenes to azobenzenes **4**.

**Scheme 1:**
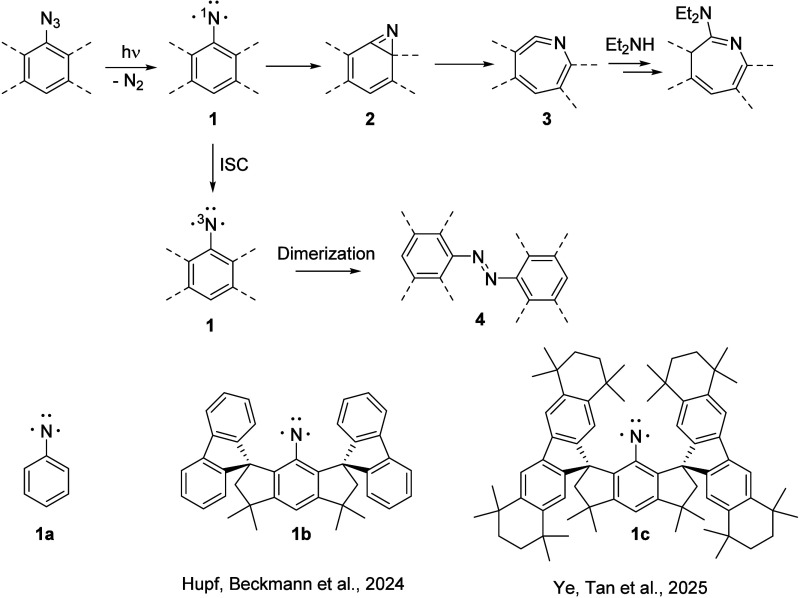
General Fate of Arylnitrenes **1** Generated Photochemically from the Corresponding Arylazide^a^

a In the singlet state, rearrangement to benzazirines **2** and didehydroazepines **3** is common, while intersystem crossing (ISC) to the triplet arylnitrene typically results in formation of azobenzenes **4**.

The barrier for formation of benzazirine **2a** from phenylnitrene is *E*_a_ = 5.6 ± 0.3 kcal/mol (A = 10^13.1 ± 0.3^ s^–1^).^7^ Consistent with theory,^8^ 2,6-dimethyl substitution increases the barrier for benzazirine formation (2,6-dimethyl phenylnitrene: *E*_a_ = 7.0 ± 0.3 kcal/mol, A = 10^13.0 ± 0.3^ s^–1^) according to laser flash photolysis (LFP).^9^ In contrast, 2,4,6-tri-*tert*-butylphenylnitrene could not be observed by LFP due to its very short lifetime, and ring opening of the benzazirine to the didehydroazepine becomes the rate-determining step (*E*_a_ = 7.4 ± 0.2 kcal/mol, A = 10^12.6 ± 0.2^ s^–1^) of the rearrangement.^10^ As the barrier for benzazirine formation is obviously quite low for 2,4,6-tri-*tert*-butylphenylnitrene, it may be expected that for the structurally related photogenerated singlet arylnitrenes **1b** and **1c** the ring enlargement should be competitive with intersystem crossing (ISC) to the triplet ground state. In this scenario, the formation of the didehydroazepine isomers **3b** and **3c** that are stable toward bimolecular degradation reactions due to the bulky substituents is anticipated. But no evidence for their formation was observed.^5,6^

Here we compare the potential energy surface (PES) for the isomerization of the arylnitrene **1b** investigated by Hupf, Beckmann, and co-workers^5^ to that of parent phenylnitrene **1a** using density functional theory (M06–2X^11^ for geometry optimization) and DLPNO–CCSD­(T)^12–14^ as implemented in Orca^15^ for single point energy evaluations (see Supporting Information for computational details). As singlet arylnitrenes are open-shell systems (**1a**: ^1^A_2_ electronic state), we give the energy of the transition states and the singlet products relative to triplet arylnitrene (**1a**: ^3^A_2_ electronic state), and employ the experimental^16,17^ triplet-singlet energy gap of **1a** (18.33 ± 0.69 kcal/mol) to fix the energy of the singlet state on the PES. In order to judge the performance of DLPNO–CCSD­(T)//M06–2X, we employed high-level computations of the complete basis set extrapolation scheme type (CBS-QB3)^18,19^ for parent phenylnitrene **1a** and its rearrangement to didehydroazepine **3a**, and also compare to previous CASPT2N//CASSCF^20^ computations.

First, we note that the CBS-QB3 and DLPNO–CCSD­(T) methods give barriers of 4–7 kcal/mol for benzazirine **2a** formation from singlet phenylnitrene **1a** via **TS1a** that are in quite good agreement with the experimental value (*E*_a_ = 5.6 ± 0.3 kcal/mol, Figure 1).^7^ Previous (symmetry-broken) DFT^21^ as well as CASPT2N^20^ approaches arrived at a too high barrier, the latter due to an overstabilization of the phenylnitrene ^1^A_2_ state.^20^ The impact of 2,6-dimethyl and 2,4,6-tri-*tert*-butyl substitution at the DLPNO–CCSD­(T) level on the barrier of benzazirine formation qualitatively follows the experimental observations: compared to parent phenylnitrene **1a**, methyl substituents *increase* the barrier for benzazirine formation by 1.2 kcal/mol (exp.^9^ by 1.8 kcal/mol) taking into account the decrease of the triplet-singlet energy gap by 1.7 kcal/mol computed earlier at the CASPT2 level.^10^ On the other hand, *tert*-butyl substituents *decrease* the barrier by 7.3 kcal/mol assuming that the singlet–triplet energy gap decreases by 2.4 kcal/mol due to 2,4,6-tri-*tert*-butyl substitution as computed earlier at the CASPT2 level.^10^ Most importantly, the nitrene could not be observed by LFP experiments, indicating a very low barrier for benzazirine formation.^10^ These data show that the DLPNO–CCSD­(T) method can qualitatively describe the impact of the substituents correctly.

**Figure 1:**
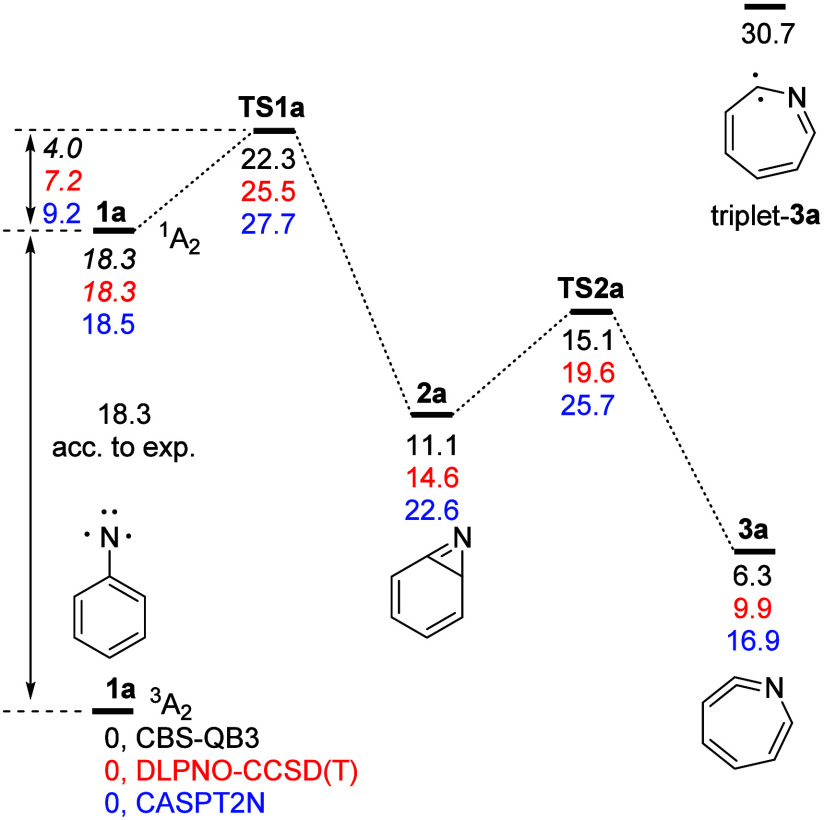
Potential energy surface for the rearrangement of phenylnitrene **1a** to didehydroazepine **3a** computed at the CBS-QB3 (black), DLPNO–CCSD­(T)/cc-pVTZ//M06–2X/6–311+G­(d,p) (red), and CASPT2N/6–311G­(2d,p)//CASSCF­(8,8)/6–31G* (blue) levels of theory. All energies are corrected for zero-point vibrational energy. The CASPT2N data were taken from Karney and Borden.^20^ Energies that rely on the experimental^16,17^ phenylnitrene singlet–triplet energy gap are given in italics.

Second, we find that didehydroazepine **3a** is higher in energy than ^3^A_2_ phenylnitrene **1a** by around 6–10 kcal/mol. This is as much as 10 kcal/mol less than the 16.9 kcal/mol obtained at the CASPT2N level.^20^ The employed extrapolation method relies on CCSD­(T) energies, and T_1_ diagnostics do not give any indication for problems in the CCSD treatment of triplet phenylnitrene **1a** (T_1_ = 0.02) or didehydroazepine **3a** (T_1_ = 0.01). We thus tend to assign a lower energy to **3a**. A consequence of this relatively lower energy of didehydroazepine **3a** is that the rearrangement of singlet phenylnitrene **1a** to didehydroazepine **3a** is more exothermic (−8 to −12 kcal/mol) than concluded previously^20^ (−1.6 kcal/mol).

The PES for parent and bulky phenylnitrene (Figure 2) was computed using the DLPNO–CCSD­(T)/cc-pVTZ//M06–2X/6–311+G­(d,p) + ZPVE method. The triplet-singlet energy gap of bulky system **1b** is expected to not differ much from that of **1a**. This seems reasonable as quite similar D values were observed by EPR spectroscopy for **1a** and **1c**.^15,22^ Indeed, symmetry-broken UM06–2X computations arrive at triplet-singlet energy gaps of 18.4 kcal/mol (exp.^16,17^ 18.3 kcal/mol) and 19.0 kcal/mol for **1a** and **1b**, respectively, further supporting the assumption of similar ST gaps. Using the UM06–2X ST gap, the barrier for the formation of the benzazirine isomer from **1b** is larger by 0.4 kcal/mol than that for parent **1a**. Although we cannot give a better estimate of the singlet–triplet energy gap, we conclude that the barrier for benzazirine formation is likely similar to that of the parent system. Both benzazirine **2b** and didehydroazepine **3b** are slightly more stable relative to the singlet nitrene **1b** than in the case of the parent, slightly increasing the thermodynamic driving force for rearrangement.

**Figure 2:**
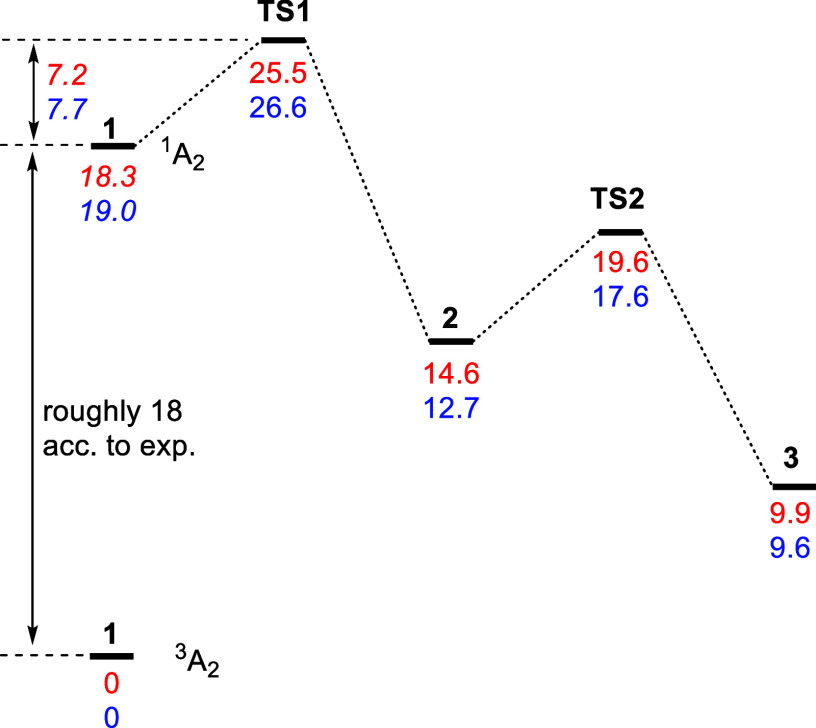
PES for the rearrangement of arylnitrenes to didehydroazepines as computed at the DLPNO–CCSD­(T)/cc-pVTZ//M06–2X/6–311+G­(d,p) + ZPVE level of theory. Red: substituent **a** (phenyl); blue: **b** (bulky). Values in italics are based on the experimental (**1a**) or computed (UM06–2X/6–311+G­(d,p); **1b**) singlet–triplet energy gap. All computed values are corrected for zero point vibrational energy.

Most importantly, there is no indication that the barriers for didehydroazepine formation are significantly higher than those for parent phenylnitrene. Hence, it is expected that formation of singlet nitrene **1b** upon photolysis should similarly result in rapid isomerization to the didehydroazepine **3b** isomer. We suggest that the photolysis be performed in the presence of small nucleophiles such as diethylamine, as this is known to be able to trap didehydroazepines readily. This would allow the identification of a rearrangement mechanism also in the case of the bulky substituted arylnitrenes **1b** and **1c**.

Should the bulky systems undergo rearrangement as predicted, then high-yield formation of the triplet nitrenes **1b** (or **1c**) needs to be accounted for. An equilibration of **3** and **1** has been invoked before to explain azobenzene formation (by ISC and triplet arylnitrene dimerization).^20,23^ Such an equilibrium lies far on the side of **3**, but with barriers of 16.8 kcal/mol (**3b**) and 15.4 kcal/mol (**3a**) formation of singlet **1** and its relaxation to the triplet state are conceivable at room temperature. It would be very interesting to run the photolysis at lower temperatures. The proposal by Schuster and co-workers^23^ of an ISC from the singlet didehydroazepine to its triplet state (an iminocarbene) must be ruled out as this is 24.4 kcal/mol (CBS-QB3) above the singlet state (triplet-**3a** in Figure 1). An alternative explanation for the experimental observation of dominant triplet arylnitrene formation could be a photophysical mechanism that involves photosensitization by the fluorene-type large π systems of the azide and direct formation of the triplet nitrene. The details pertaining to such a process were investigated computationally for phenyl azide by Soto and Otero.^24^

In summary, the computations show that the rearrangements of the singlet arylnitrenes **1a** and **1b** share similar PES. This suggests that the photolysis of the corresponding aryl azide could result in fast formation of the didehydroazepine isomer after N_2_ extrusion, like the case of phenyl azide, provided that singlet phenylnitrene is involved as a reactive intermediate. We suggest that trapping experiments, e.g. with diethylamine, should be done to investigate the possible formation of the didehydroazepine. The computed PESs indicate that the equilibration of didehydroazepine and singlet arylnitrene is in principle feasible at room temperature. An alternative explanation for the formation of triplet nitrene could be an intramolecular photosensitization involving the organic π system of the fluorene units that ultimately avoids population of the singlet nitrene state.

## Computational Methods

All structures were fully optimized using the M06–2X functional in conjunction with the 6–311+G­(d,p) basis set.^11^ Computation of harmonic vibrational frequencies confirmed the nature of stationary points as minima or transition states. For singlet nitrenes and **TS1** the spin-symmetry-broken approach was used. In addition, the CBS-QB3 ^18,19^ method was employed to compute the energy of stationary points for the rearrangement of parent phenylnitrene. The CBS-QB3 geometry and vibrational frequencies of **TS1a** were computed using the spin-unrestricted UB3LYP method, while the higher-level computations were done using the spin-restricted approach. The M06–2X and CBS-QB3 computations were performed using Gaussian 16.^25^ The M06–2X/6–311+G­(d,p) structures were employed for single point energy evaluation using the domain based local pair-natural orbital (DLPNO) approximation (with TightPNO setting along with the “usefullLMP2guess false” option) to coupled-cluster with singles, doubles, and a perturbative estimate of triples excitations, DLPNO–CCSD­(T),^12–14^ as implemented in Orca 6 ^15^ using the spin-restricted approach for all species except for triplet nitrenes that were treated with spin-unrestricted approximation. The cc-pVTZ^26^ basis set along with the recommended RI and RIJK fitting bases was used.^27,28^ The DLPNO–CCSD­(T)/cc-pVTZ energies were corrected for zero-point vibrational energies computed at M06–2X/6–311+G­(d,p).

## Supplementary Material



## Data Availability

The data underlying this study are available in the published article and its Supporting Information.

## Notes

Cartesian coordinates of all optimized minima and transition states. (PDF)
