# Supplementary material for: Impact of Bulky Substituents on the Singlet Arylnitrene Ring Enlargement
Source: J Org Chem. 2025 Jul 28;90(31):11378–81. doi: 10.1021/acs.joc.5c00720 (PMC12340949; doi:10.1021/acs.joc.5c00720)
Supplement: Supplementary file 1 [file jo5c00720_si_001.pdf]

Supporting Information

Impact of Bulky Substituents on the Singlet Arylnitrene Ring Enlargement

Holger F. Bettinger

Institut für Organische Chemie, Eberhard Karls Universität Tübingen, Auf der Morgenstelle 18, 72076 Tübingen, Germany

holger.bettinger@uni-tuebingen.de

Table of Contents

Cartesian Coordinates S2

Cartesian Coordinates

All Cartesian coordinates are given in Å.

*A. M06-2X/6-311+G** Geometries*

I. Phenylnitrene **1a** PES

```
12
Triplet 1a, E(UM062X) =  -286.24935
6        0.000000000      1.226433000      0.337382000
6        0.000000000      0.000000000      1.058879000
6        0.000000000     -1.226433000      0.337382000
6        0.000000000     -1.212143000     -1.042842000
6        0.000000000      1.212143000     -1.042842000
6        0.000000000      0.000000000     -1.739051000
1        0.000000000      0.000000000     -2.822027000
7        0.000000000      0.000000000      2.394001000
1        0.000000000      2.147533000     -1.589392000
1        0.000000000      2.155081000      0.894681000
1        0.000000000     -2.155081000      0.894681000
1        0.000000000     -2.147533000     -1.589392000
```

```
12
Singlet 1a, E(UM062X) =  -286.22047
6        0.337256000      1.222137000      0.000069000
6        1.057815000      0.000000000     -0.000358000
6        0.337256000     -1.222137000      0.000069000
6       -1.046025000     -1.211198000      0.000047000
6       -1.046025000      1.211198000      0.000047000
6       -1.738098000      0.000000000     -0.000103000
1       -2.821567000      0.000000000     -0.000031000
7        2.399912000      0.000000000      0.000088000
1        0.897268000      2.149070000      0.000242000
1       -1.592717000      2.146457000      0.000152000
1       -1.592717000     -2.146457000      0.000153000
1        0.897268000     -2.149070000      0.000242000
```

```
12
TS1a, i806 cm-1, E(UM062X) =  -286.20174
6        0.641414000     -1.038954000     -0.370974000
6        1.034282000      0.349701000     -0.147270000
6        0.040004000      1.410746000     -0.197733000
6       -1.239724000      1.014511000      0.003589000
6       -0.670344000     -1.372583000      0.026178000
6       -1.585577000     -0.365000000      0.220843000
1       -2.613747000     -0.608178000      0.458395000
7        2.090297000      0.083335000      0.481754000
1        0.325536000      2.442002000     -0.356977000
1       -2.033452000      1.752538000      0.017523000
1       -0.983609000     -2.409995000      0.056239000
1        1.352864000     -1.750237000     -0.755249000
```

```
12
2a, E(RM062X) =  -286.23064
6       -1.154954000      0.513169000     -0.413676000
6       -0.700821000     -0.868603000     -0.109457000
6        0.627764000     -1.394715000     -0.214081000
6        1.527106000     -0.410912000      0.035908000
6       -0.097783000      1.453232000     -0.069811000
6        1.144487000      0.978471000      0.221684000
1        1.933857000      1.677147000      0.472043000
7       -1.619074000     -0.498073000      0.650843000
1        0.875248000     -2.418988000     -0.451721000
1        2.583691000     -0.652551000      0.066483000
1       -0.259057000      2.523293000     -0.154544000
1       -1.875018000      0.733764000     -1.191569000
```

12
TS2a, i465,5 cm-1, E(RM062X) = -286.22283

6	1.308189000	0.469395000	0.324771000
6	0.518948000	-1.068236000	0.027232000
6	-0.799919000	-1.300253000	0.310560000
6	-1.562321000	-0.192144000	-0.081878000
6	0.297979000	1.397526000	0.140366000
6	-1.009008000	1.077270000	-0.269814000
1	-1.685215000	1.891141000	-0.499513000
7	1.483545000	-0.673537000	-0.627560000
1	0.478407000	2.404891000	0.505025000
1	2.117317000	0.604588000	1.031779000
1	-1.179067000	-2.184791000	0.797480000
1	-2.639467000	-0.302421000	-0.149278000

12
3a, E(RM062X) = -286.23609

6	1.586530000	0.121265000	0.149263000
6	-0.011319000	-1.288544000	-0.037083000
6	-1.232023000	-1.002283000	0.359677000
6	-1.566791000	0.340424000	-0.149815000
6	0.777243000	1.196047000	0.241888000
6	-0.621200000	1.306325000	-0.185207000
1	-0.920375000	2.291243000	-0.530624000
7	1.124230000	-1.080670000	-0.476296000
1	-2.574131000	0.559024000	-0.492421000
1	-1.833975000	-1.582270000	1.041510000
1	1.236173000	2.113166000	0.596788000
1	2.628067000	0.144118000	0.446487000

II. Arylnitrene **1b** PES

74
Triplet 1b, E(UM062X) = -1598.58165

6	-0.359647000	-0.000043000	2.614012000
6	-0.359647000	-0.000043000	-2.614012000
6	0.573448000	-1.177158000	-2.865333000
6	0.573448000	-1.177158000	2.865333000
6	-0.936058000	-0.000051000	1.215577000
6	1.849282000	-0.735119000	-3.234608000
6	-0.185193000	0.000006000	0.000000000
6	-0.936058000	-0.000051000	-1.215577000
6	-2.316940000	-0.000117000	-1.204064000
6	-2.316940000	-0.000117000	1.204064000
6	2.859144000	-1.648927000	3.511544000
1	3.850637000	-1.313279000	3.793991000
6	-3.028919000	-0.000153000	0.000000000
1	-4.115451000	-0.000195000	0.000000000
6	0.573293000	1.177182000	-2.865415000
6	-1.637400000	-0.000278000	3.516886000
1	-1.630414000	-0.879197000	4.164062000
1	-1.630461000	0.878212000	4.164646000
6	1.849282000	-0.735119000	3.234608000
6	0.293908000	-2.528765000	2.759771000
1	-0.691835000	-2.868304000	2.456485000
6	0.573293000	1.177182000	2.865415000
6	1.849186000	0.735264000	-3.234663000
6	1.306411000	-3.446862000	3.037410000
1	1.107830000	-4.509021000	2.957183000
6	-1.637400000	-0.000278000	-3.516886000
1	-1.630461000	0.878212000	-4.164646000
1	-1.630414000	-0.879197000	-4.164062000
6	2.859144000	-1.648927000	-3.511544000
1	3.850637000	-1.313279000	-3.793991000
6	1.849186000	0.735264000	3.234663000
6	-2.899791000	-0.000094000	2.604100000
6	-2.899791000	-0.000094000	-2.604100000
6	2.858952000	1.649155000	3.511665000
1	3.850479000	1.313583000	3.794085000
6	2.576375000	-3.008741000	-3.411326000
1	3.352475000	-3.735436000	-3.620337000
6	1.306411000	-3.446862000	-3.037410000
1	1.107830000	-4.509021000	-2.957183000
6	1.306046000	3.446951000	3.037630000
1	1.107367000	4.509096000	2.957455000

6	2.576375000	-3.008741000	3.411326000
1	3.352475000	-3.735436000	3.620337000
6	0.293908000	-2.528765000	-2.759771000
1	-0.691835000	-2.868304000	-2.456485000
6	0.293636000	2.528770000	2.759929000
1	-0.692114000	2.868260000	2.456638000
6	0.293636000	2.528770000	-2.759929000
1	-0.692114000	2.868260000	-2.456638000
6	2.576050000	3.008945000	3.411533000
1	3.352075000	3.735707000	3.620589000
6	2.858952000	1.649155000	-3.511665000
1	3.850479000	1.313583000	-3.794085000
6	-3.754210000	-1.252759000	-2.834509000
1	-4.133056000	-1.271376000	-3.860148000
1	-4.611460000	-1.273266000	-2.155938000
1	-3.167009000	-2.159174000	-2.668267000
6	-3.753977000	1.252724000	2.834535000
1	-4.611190000	1.273418000	2.155922000
1	-3.166645000	2.159061000	2.668365000
1	-4.132874000	1.271351000	3.860155000
6	-3.753977000	1.252724000	-2.834535000
1	-3.166645000	2.159061000	-2.668365000
1	-4.611190000	1.273418000	-2.155922000
1	-4.132874000	1.271351000	-3.860155000
6	-3.754210000	-1.252759000	2.834509000
1	-4.133056000	-1.271376000	3.860148000
1	-3.167009000	-2.159174000	2.668267000
1	-4.611460000	-1.273266000	2.155938000
6	2.576050000	3.008945000	-3.411533000
1	3.352075000	3.735707000	-3.620589000
6	1.306046000	3.446951000	-3.037630000
1	1.107367000	4.509096000	-2.957455000
7	1.142858000	0.000125000	0.000000000

74
Singlet 1b, E(UM062X) = -1598.55145

6	-2.609167000	-0.356780000	0.049230000
6	2.609168000	-0.356780000	0.049229000
6	2.855519000	0.647366000	1.166647000
6	-2.855518000	0.647366000	1.166647000
6	-1.210314000	-0.932151000	0.070465000
6	3.238311000	1.890031000	0.649084000
6	0.000000000	-0.185162000	0.048765000
6	1.210315000	-0.932151000	0.070464000
6	1.201575000	-2.317022000	0.104623000
6	-1.201574000	-2.317022000	0.104624000
6	-3.515612000	2.952737000	1.500563000
1	-3.808121000	3.919493000	1.106463000
6	0.000001000	-3.025193000	0.120738000
1	0.000001000	-4.111893000	0.146211000
6	2.870106000	0.500167000	-1.183235000
6	-3.511591000	-1.631326000	0.145697000
1	-4.080592000	-1.600383000	1.076698000
1	-4.232233000	-1.643824000	-0.673935000
6	-3.238312000	1.890031000	0.649083000
6	-2.738691000	0.452152000	2.532080000
1	-2.427508000	-0.509336000	2.929631000
6	-2.870107000	0.500166000	-1.183235000
6	3.247948000	1.798187000	-0.818052000
6	-3.015543000	1.518154000	3.387684000
1	-2.925532000	1.386644000	4.459438000
6	3.511592000	-1.631325000	0.145695000
1	4.232234000	-1.643823000	-0.673937000
1	4.080593000	-1.600383000	1.076697000
6	3.515610000	2.952737000	1.500565000
1	3.808118000	3.919494000	1.106464000
6	-3.247949000	1.798185000	-0.818052000
6	-2.603346000	-2.896322000	0.111136000
6	2.603347000	-2.896321000	0.111134000
6	-3.538101000	2.748041000	-1.789875000
1	-3.826552000	3.755873000	-1.513158000
6	3.401883000	2.755894000	2.874453000
1	3.610308000	3.574423000	3.553179000
6	3.015544000	1.518153000	3.387684000
1	2.925533000	1.386641000	4.459439000
6	-3.058136000	1.093934000	-3.492704000
1	-2.979963000	0.831875000	-4.541167000

6	-3.401884000	2.755895000	2.874452000
1	-3.610310000	3.574424000	3.553178000
6	2.738692000	0.452150000	2.532080000
1	2.427511000	-0.509338000	2.929630000
6	-2.766900000	0.141437000	-2.516215000
1	-2.453397000	-0.858616000	-2.798663000
6	2.766900000	0.141438000	-2.516215000
1	2.453398000	-0.858615000	-2.798664000
6	-3.442305000	2.384405000	-3.130767000
1	-3.661772000	3.113166000	-3.902024000
6	3.538099000	2.748043000	-1.789874000
1	3.826549000	3.755876000	-1.513156000
6	2.826986000	-3.772458000	1.349522000
1	3.857639000	-4.136984000	1.376080000
1	2.161641000	-4.640237000	1.342650000
1	2.638585000	-3.205441000	2.264517000
6	-2.846745000	-3.728243000	-1.154637000
1	-2.160883000	-4.578698000	-1.200838000
1	-2.699848000	-3.124662000	-2.053191000
1	-3.869509000	-4.115057000	-1.164222000
6	2.846746000	-3.728242000	-1.154639000
1	2.699849000	-3.124659000	-2.053193000
1	2.160884000	-4.578696000	-1.200841000
1	3.869510000	-4.115056000	-1.164225000
6	-2.826984000	-3.772458000	1.349525000
1	-3.857637000	-4.136985000	1.376083000
1	-2.638584000	-3.205441000	2.264519000
1	-2.161639000	-4.640236000	1.342653000
6	3.442303000	2.384408000	-3.130766000
1	3.661770000	3.113169000	-3.902022000
6	3.058136000	1.093936000	-3.492704000
1	2.979963000	0.831878000	-4.541167000
7	0.000000000	1.151294000	0.006496000

74

TS1b, i761.9 cm-1, E(UM062X) = -1598.52982

6	2.496050000	0.327902000	-0.427656000
6	-2.562548000	0.303614000	0.215099000
6	-2.274183000	-0.868978000	1.144143000
6	2.422668000	-0.230119000	0.996824000
6	1.159239000	0.948131000	-0.752550000
6	-2.796394000	-2.059209000	0.623872000
6	-0.152790000	0.334449000	-0.924912000
6	-1.282378000	0.942491000	-0.238719000
6	-1.159892000	2.253168000	0.081245000
6	1.189372000	2.347259000	-0.563311000
6	2.859648000	-2.275397000	2.219917000
1	3.187015000	-3.308714000	2.247357000
6	0.041560000	3.005206000	-0.174893000
1	0.066081000	4.063920000	0.065714000
6	-3.311173000	-0.387318000	-0.914977000
6	3.461321000	1.550161000	-0.571870000
1	4.197747000	1.549860000	0.233834000
1	4.004542000	1.451775000	-1.513840000
6	2.849718000	-1.563669000	1.025923000
6	1.968858000	0.392402000	2.148810000
1	1.587225000	1.408537000	2.116738000
6	2.963107000	-0.889570000	-1.201871000
6	-3.450926000	-1.757153000	-0.658553000
6	1.983632000	-0.320394000	3.348708000
1	1.637275000	0.153162000	4.260497000
6	-3.312773000	1.509007000	0.870419000
1	-4.267328000	1.669147000	0.365532000
1	-3.531633000	1.280596000	1.915196000
6	-2.615435000	-3.261358000	1.298945000
1	-3.014240000	-4.187978000	0.901540000
6	3.199952000	-1.968591000	-0.343988000
6	2.617937000	2.863997000	-0.581925000
6	-2.410803000	2.778580000	0.761812000
6	3.673117000	-3.178975000	-0.840530000
1	3.856353000	-4.018152000	-0.178843000
6	-1.894291000	-3.256871000	2.490255000
1	-1.737311000	-4.186015000	3.025282000
6	-1.356536000	-2.072929000	2.995027000
1	-0.776731000	-2.091930000	3.910228000
6	3.658602000	-2.218041000	-3.063047000
1	3.829677000	-2.330734000	-4.126971000

6	2.430300000	-1.640336000	3.383071000
1	2.432758000	-2.181129000	4.322384000
6	-1.543975000	-0.867001000	2.320764000
1	-1.105658000	0.051081000	2.700258000
6	3.185666000	-1.007138000	-2.563643000
1	2.969149000	-0.180022000	-3.231525000
6	-3.809604000	0.171088000	-2.080041000
1	-3.679570000	1.228984000	-2.285976000
6	3.903203000	-3.293603000	-2.207560000
1	4.269280000	-4.228621000	-2.614852000
6	-4.108715000	-2.580586000	-1.565425000
1	-4.219254000	-3.642118000	-1.374592000
6	-2.074824000	3.343079000	2.148070000
1	-2.987087000	3.661776000	2.659808000
1	-1.410859000	4.208477000	2.072047000
1	-1.581480000	2.587066000	2.764703000
6	2.860178000	3.645085000	-1.882433000
1	2.241354000	4.545906000	-1.913793000
1	2.613088000	3.030598000	-2.751620000
1	3.909770000	3.944494000	-1.955648000
6	-3.073732000	3.866645000	-0.091193000
1	-3.323119000	3.482781000	-1.083268000
1	-2.410039000	4.726749000	-0.216782000
1	-3.993744000	4.215961000	0.385688000
6	2.939002000	3.760742000	0.616952000
1	3.979420000	4.092981000	0.567943000
1	2.797418000	3.230686000	1.560995000
1	2.305432000	4.651604000	0.622734000
6	-4.618964000	-2.016235000	-2.731396000
1	-5.132193000	-2.643113000	-3.450927000
6	-4.469712000	-0.653702000	-2.989738000
1	-4.863823000	-0.236108000	-3.908480000
7	0.092800000	-0.265216000	-2.001300000

74
2, E(RM062X) = -1598.56434

6	-2.569755000	0.154624000	0.416298000
6	2.414988000	0.396113000	-0.288802000
6	2.253084000	-0.951916000	-0.977032000
6	-2.320729000	-0.368807000	-0.992136000
6	-1.337790000	0.888213000	0.946399000
6	3.036001000	-1.930046000	-0.352563000
6	0.061025000	0.465808000	1.011690000
6	1.106091000	0.968280000	0.177317000
6	0.837603000	2.226173000	-0.258613000
6	-1.471670000	2.292030000	0.564418000
6	-2.299351000	-2.457778000	-2.225445000
1	-2.416336000	-3.535302000	-2.260737000
6	-0.407492000	2.929390000	0.007017000
1	-0.511936000	3.941776000	-0.373707000
6	3.354454000	0.036935000	0.855610000
6	-3.649933000	1.285117000	0.460636000
1	-4.261260000	1.252941000	-0.443080000
1	-4.312201000	1.117771000	1.312668000
6	-2.474467000	-1.761612000	-1.034693000
6	-1.991284000	0.338465000	-2.139048000
1	-1.860464000	1.415442000	-2.108467000
6	-2.910870000	-1.114831000	1.161060000
6	3.727762000	-1.311611000	0.788725000
6	-1.826931000	-0.359071000	-3.337226000
1	-1.578790000	0.180510000	-4.244359000
6	2.937057000	1.539748000	-1.222816000
1	3.959176000	1.812291000	-0.953930000
1	2.955129000	1.175672000	-2.251755000
6	3.036582000	-3.238602000	-0.822193000
1	3.640304000	-4.001498000	-0.343486000
6	-2.841130000	-2.227149000	0.312224000
6	-2.933216000	2.674192000	0.605801000
6	1.975973000	2.763024000	-1.107547000
6	-3.099918000	-3.504674000	0.795831000
1	-3.048811000	-4.368635000	0.142686000
6	2.234632000	-3.555325000	-1.915942000
1	2.219853000	-4.570871000	-2.293800000
6	1.439289000	-2.583215000	-2.522307000
1	0.804386000	-2.849882000	-3.358801000
6	-3.479293000	-2.548197000	2.989015000
1	-3.719765000	-2.687439000	4.036254000

6	-1.979190000	-1.744573000	-3.378472000
1	-1.851777000	-2.269798000	-4.318228000
6	1.441956000	-1.269704000	-2.053274000
1	0.804656000	-0.519718000	-2.511816000
6	-3.218220000	-1.267747000	2.503064000
1	-3.238874000	-0.409165000	3.166094000
6	3.828325000	0.851449000	1.870375000
1	3.515986000	1.888149000	1.940396000
6	-3.423110000	-3.654833000	2.141681000
1	-3.626818000	-4.642539000	2.538118000
6	4.598960000	-1.851435000	1.727961000
1	4.890214000	-2.894769000	1.680917000
6	1.456469000	3.180648000	-2.489875000
1	2.289493000	3.481377000	-3.131279000
1	0.766404000	4.025423000	-2.416852000
1	0.933073000	2.349337000	-2.970545000
6	-3.236154000	3.265316000	1.994080000
1	-2.707299000	4.211059000	2.135549000
1	-2.920579000	2.576512000	2.782154000
1	-4.309915000	3.445644000	2.099938000
6	2.650905000	3.960465000	-0.428578000
1	3.048514000	3.683437000	0.549857000
1	1.941073000	4.779982000	-0.286219000
1	3.476508000	4.329542000	-1.043365000
6	-3.349548000	3.662648000	-0.481136000
1	-4.422296000	3.863782000	-0.419904000
1	-3.133274000	3.271039000	-1.478032000
1	-2.826172000	4.615610000	-0.364322000
6	5.086111000	-1.027248000	2.739076000
1	5.763965000	-1.431248000	3.481625000
6	4.701933000	0.311090000	2.813800000
1	5.079846000	0.933455000	3.616103000
7	-0.499558000	0.437428000	2.137722000

74

TS2, i350.6 cm-1, E(RM062X) = -1598.55543018

6	-2.579547000	0.136891000	-0.342819000
6	2.496024000	0.444103000	0.480254000
6	2.671672000	-0.188819000	-0.896726000
6	-1.902947000	-1.033025000	-1.040043000
6	-1.589842000	0.912221000	0.506818000
6	3.243406000	-1.462741000	-0.785622000
6	-0.087592000	0.412874000	1.141844000
6	1.107920000	0.963055000	0.750787000
6	0.981019000	2.342314000	0.568310000
6	-1.415254000	2.238101000	0.100516000
6	-1.991279000	-3.449434000	-1.162331000
1	-2.439278000	-4.395151000	-0.878232000
6	-0.242773000	2.975370000	0.314380000
1	-0.229652000	4.028565000	0.051297000
6	2.960299000	-0.679149000	1.388738000
6	-3.099088000	1.249471000	-1.306566000
1	-2.740162000	1.034546000	-2.315118000
1	-4.190360000	1.254481000	-1.339582000
6	-2.488815000	-2.247677000	-0.669806000
6	-0.808954000	-1.000883000	-1.888139000
1	-0.340863000	-0.057318000	-2.152808000
6	-3.648288000	-0.578661000	0.473296000
6	3.410417000	-1.774478000	0.642593000
6	-0.302314000	-2.204532000	-2.376699000
1	0.563680000	-2.196001000	-3.027340000
6	3.325570000	1.765352000	0.646555000
1	3.805803000	1.745113000	1.627006000
1	4.114375000	1.818871000	-0.105905000
6	3.556603000	-2.200652000	-1.922282000
1	4.003334000	-3.185192000	-1.839558000
6	-3.587329000	-1.963338000	0.265284000
6	-2.538697000	2.631546000	-0.846021000
6	2.359684000	2.988147000	0.564084000
6	-4.472528000	-2.820240000	0.909011000
1	-4.424808000	-3.891354000	0.747323000
6	3.290452000	-1.649570000	-3.173146000
1	3.535095000	-2.206914000	-4.069887000
6	2.699573000	-0.390495000	-3.283649000
1	2.482812000	0.016343000	-4.264601000
6	-5.476938000	-0.901615000	1.987163000
1	-6.214565000	-0.495064000	2.668803000

6	-0.893249000	-3.416689000	-2.018105000
1	-0.486054000	-4.343848000	-2.404052000
6	2.380822000	0.344872000	-2.141722000
1	1.900061000	1.313898000	-2.227803000
6	-4.587006000	-0.044405000	1.340113000
1	-4.629786000	1.022264000	1.526959000
6	2.974641000	-0.710656000	2.773325000
1	2.607536000	0.133342000	3.349364000
6	-5.421409000	-2.277435000	1.770782000
1	-6.119626000	-2.929283000	2.282397000
6	3.895084000	-2.912057000	1.278308000
1	4.244577000	-3.763901000	0.705809000
6	2.605135000	3.840245000	-0.686569000
1	3.604246000	4.282447000	-0.645630000
1	1.881210000	4.656078000	-0.752402000
1	2.535826000	3.247900000	-1.599980000
6	-3.595888000	3.445926000	-0.086973000
1	-3.171884000	4.390019000	0.265492000
1	-3.964288000	2.901469000	0.783519000
1	-4.443458000	3.671190000	-0.740886000
6	2.512448000	3.873632000	1.809407000
1	2.306115000	3.300805000	2.716634000
1	1.818275000	4.717241000	1.773588000
1	3.530965000	4.268360000	1.870762000
6	-2.047823000	3.438471000	-2.050013000
1	-2.865926000	3.592224000	-2.759287000
1	-1.237918000	2.914470000	-2.564414000
1	-1.680585000	4.422203000	-1.746811000
6	3.915605000	-2.940616000	2.670141000
1	4.285717000	-3.819872000	3.183884000
6	3.456517000	-1.852394000	3.412425000
1	3.470427000	-1.898111000	4.494809000
7	-1.128975000	0.375999000	1.812177000

74

3b, E(RM062X) = -1598.56320

6	2.653478000	0.305338000	0.240597000
6	-2.750925000	0.305475000	-0.417294000
6	-2.562610000	-0.248359000	0.990230000
6	2.273592000	-1.081218000	0.747505000
6	1.479670000	0.947545000	-0.443296000
6	-2.889258000	-1.609152000	1.034370000
6	-0.301013000	0.363207000	-1.397546000
6	-1.434775000	0.837824000	-0.931837000
6	-1.348186000	2.267548000	-0.647551000
6	1.004917000	2.063491000	0.154258000
6	2.953493000	-3.396651000	0.536335000
1	3.609664000	-4.156315000	0.126282000
6	-0.212238000	2.814795000	-0.151739000
1	-0.231852000	3.855066000	0.164165000
6	-3.280671000	-0.907881000	-1.154606000
6	2.990808000	1.350862000	1.346067000
1	3.037531000	0.866934000	2.323183000
1	3.974301000	1.780992000	1.147488000
6	3.126141000	-2.055384000	0.213448000
6	1.219875000	-1.434768000	1.574654000
1	0.526498000	-0.688414000	1.951775000
6	3.810157000	-0.020097000	-0.689080000
6	-3.345310000	-2.018831000	-0.303693000
6	1.045663000	-2.780977000	1.897471000
1	0.223568000	-3.071096000	2.540957000
6	-3.648926000	1.591987000	-0.460656000
1	-4.412959000	1.481502000	-1.232901000
1	-4.163392000	1.708352000	0.495075000
6	-2.755494000	-2.328178000	2.217151000
1	-3.004661000	-3.382892000	2.257769000
6	4.088798000	-1.391956000	-0.679548000
6	1.899025000	2.463078000	1.322070000
6	-2.748638000	2.838922000	-0.762638000
6	5.125445000	-1.906608000	-1.450072000
1	5.347128000	-2.967975000	-1.447360000
6	-2.293844000	-1.668133000	3.354094000
1	-2.184663000	-2.212476000	4.284960000
6	-1.974592000	-0.310633000	3.309111000
1	-1.618841000	0.186741000	4.204312000
6	5.589341000	0.333993000	-2.246946000
1	6.177898000	0.997477000	-2.869352000

6	1.909575000	-3.750114000	1.387922000
1	1.758309000	-4.791463000	1.647006000
6	-2.108230000	0.410608000	2.121785000
1	-1.846852000	1.463842000	2.082290000
6	4.551235000	0.848980000	-1.471242000
1	4.321976000	1.910134000	-1.487694000
6	-3.647207000	-1.013480000	-2.485777000
1	-3.581609000	-0.155660000	-3.147569000
6	5.873958000	-1.031364000	-2.232740000
1	6.684334000	-1.414634000	-2.841507000
6	-3.785898000	-3.248675000	-0.779562000
1	-3.836130000	-4.112552000	-0.126344000
6	-3.010357000	3.986667000	0.210370000
1	-4.053050000	4.307958000	0.144909000
1	-2.381268000	4.849683000	-0.023057000
1	-2.809707000	3.681828000	1.240923000
6	2.529236000	3.842029000	1.092757000
1	1.770622000	4.628583000	1.055489000
1	3.087407000	3.859913000	0.153397000
1	3.217726000	4.082228000	1.907985000
6	-2.979688000	3.308767000	-2.207688000
1	-2.778881000	2.497538000	-2.912278000
1	-2.319082000	4.143882000	-2.452365000
1	-4.016329000	3.632202000	-2.338453000
6	1.095879000	2.477426000	2.629770000
1	1.745362000	2.733434000	3.471731000
1	0.652614000	1.496883000	2.823110000
1	0.288484000	3.214743000	2.586163000
6	-4.161037000	-3.351882000	-2.116267000
1	-4.506995000	-4.302189000	-2.505134000
6	-4.092253000	-2.245738000	-2.963180000
1	-4.383361000	-2.347318000	-4.001834000
7	0.910197000	0.410328000	-1.649310000

III. 2,6-Dimethylphenylnitrene **1c** PES

18
Triplet 1c, E(UM062X) = -364.86811

6	-1.246313000	0.035228000	0.000002000
6	-0.000002000	0.748873000	0.000004000
6	1.246312000	0.035237000	-0.000022000
6	1.213252000	-1.346114000	-0.000016000
6	-1.213246000	-1.346121000	0.000013000
6	0.000006000	-2.038819000	0.000005000
1	0.000007000	-3.122084000	0.000017000
7	-0.000010000	2.074306000	0.000003000
1	-2.147687000	-1.896627000	0.000036000
1	2.147698000	-1.896611000	-0.000025000
6	2.530444000	0.811906000	0.000007000
1	2.590139000	1.459896000	0.877931000
1	3.389311000	0.140901000	-0.000390000
1	2.589829000	1.460538000	-0.877457000
6	-2.530445000	0.811897000	-0.000007000
1	-2.590004000	1.460180000	0.877707000
1	-2.589958000	1.460244000	-0.877678000
1	-3.389316000	0.140897000	-0.000072000

18
Singlet 1c, E(UM062X) = -364.83903

6	-1.242331000	0.038525000	0.000045000
6	-0.000003000	0.754205000	0.000079000
6	1.242331000	0.038535000	0.000008000
6	1.212255000	-1.346786000	-0.000011000
6	-1.212247000	-1.346797000	0.000037000
6	0.000006000	-2.033789000	0.000005000
1	0.000010000	-3.117782000	0.000019000
7	-0.000013000	2.085205000	0.000090000
1	-2.145668000	-1.898818000	0.000076000
1	2.145680000	-1.898800000	-0.000021000
6	2.533087000	0.803341000	-0.000069000
1	2.595818000	1.451308000	0.876818000
1	3.385455000	0.123983000	-0.000950000
1	2.595036000	1.452503000	-0.876131000
6	-2.533087000	0.803331000	-0.000088000
1	-2.595508000	1.451930000	0.876346000

```
1       -2.595337000      1.451871000     -0.876597000
1       -3.385459000      0.123978000     -0.000230000
```

```
18
TS1c, i712.7 cm-1, -364.82075
6        1.226359000     -0.090816000      0.091634000
6       -0.051435000     -0.769619000     -0.100911000
6       -1.333089000     -0.102362000      0.129730000
6       -1.276628000      1.249904000      0.066855000
6        1.172904000      1.304579000     -0.182030000
6       -0.038500000      1.944295000     -0.178493000
1       -0.073687000      3.020068000     -0.305633000
7        0.359447000     -1.642899000     -0.909368000
1       -2.188031000      1.830812000      0.162926000
1        2.092802000      1.875072000     -0.264030000
6        2.492196000     -0.763377000      0.517828000
1        3.225000000     -0.795783000     -0.292248000
1        2.935282000     -0.200962000      1.345441000
1        2.293417000     -1.785129000      0.834640000
6       -2.559592000     -0.932821000      0.300156000
1       -2.603053000     -1.672017000     -0.507621000
1       -2.529325000     -1.488750000      1.240723000
1       -3.461821000     -0.321714000      0.282761000
```

```
18
2c, E(RM062X) =  -364.84854
6       -1.252167000     -0.229536000      0.054247000
6        0.110535000     -0.734143000      0.323049000
6        1.381991000     -0.179914000     -0.066112000
6        1.256233000      1.170428000     -0.130484000
6       -1.214295000      1.234689000      0.081401000
6       -0.010947000      1.864553000      0.045323000
1        0.020485000      2.948057000      0.042386000
7       -0.671195000     -0.961766000      1.276479000
1        2.138773000      1.769249000     -0.333247000
1       -2.132360000      1.811319000      0.008820000
6       -2.295780000     -0.948139000     -0.758147000
1       -2.276360000     -0.629403000     -1.803132000
1       -2.126468000     -2.024715000     -0.716581000
1       -3.293240000     -0.747526000     -0.356996000
6        2.618485000     -0.981329000     -0.319350000
1        3.456738000     -0.324157000     -0.551447000
1        2.872591000     -1.585649000      0.555068000
1        2.473876000     -1.664470000     -1.159786000
```

IV. 2,4,6-Tri(tert-butyl)phenylnitrene **1d** PES

```
48
Triplet 1d, E(UM062X) =  -757.90485
6        1.408741000      0.241524000      0.000038000
6        0.810284000     -1.078781000     -0.000052000
6       -0.626070000     -1.216513000      0.000092000
6       -1.379979000     -0.055252000      0.000066000
6        0.568993000      1.340402000      0.000087000
6       -0.827475000      1.227898000      0.000043000
7        1.607486000     -2.140940000     -0.000377000
1        1.006910000      2.326524000      0.000153000
1       -2.459653000     -0.147739000      0.000028000
6       -1.385296000     -2.554258000      0.000044000
6       -2.273689000     -2.615968000     -1.257813000
6       -2.274905000     -2.615525000      1.257066000
1       -1.663871000     -2.565304000      2.161547000
1       -2.998444000     -1.798866000      1.290726000
1       -2.829714000     -3.557621000      1.269800000
1       -2.997096000     -1.799221000     -1.292407000
1       -2.828554000     -3.558032000     -1.270758000
1       -1.661779000     -2.566094000     -2.161721000
6        2.933698000      0.400468000      0.000049000
6        3.536100000     -0.246114000     -1.262198000
6        3.536148000     -0.246577000      1.262032000
1        3.108190000      0.201818000      2.162660000
1        3.358656000     -1.321297000      1.291881000
1        4.616500000     -0.077385000      1.277257000
1        3.358635000     -1.320828000     -1.292416000
```

```
1        4.616448000     -0.076887000     -1.277425000
1        3.108077000      0.202587000     -2.162643000
6       -0.506099000     -3.810904000      0.000770000
1        0.131397000     -3.864850000      0.884301000
1       -1.161132000     -4.686808000      0.000980000
1        0.131820000     -3.865605000     -0.882401000
6        3.350129000      1.877614000      0.000319000
1        2.990420000      2.402855000     -0.888225000
1        2.990417000      2.402525000      0.889059000
1        4.440689000      1.935843000      0.000329000
6       -1.754545000      2.443357000     -0.000015000
6       -2.642977000      2.403830000      1.256042000
6       -2.642843000      2.403759000     -1.256167000
1       -2.031419000      2.426617000     -2.161562000
1       -3.259229000      1.502953000     -1.287376000
1       -3.310893000      3.269479000     -1.268912000
1       -3.259304000      1.502985000      1.287282000
1       -3.311109000      3.269489000      1.268625000
1       -2.031665000      2.426834000      2.161508000
6       -0.982237000      3.766528000     -0.000008000
1       -0.352129000      3.865658000      0.887530000
1       -0.352041000      3.865591000     -0.887487000
1       -1.690397000      4.598430000     -0.000064000


48
Singlet 1d, E(UM062X) =  -757.87517
6        1.414591000      0.200442000     -0.000004000
6        0.785933000     -1.102929000      0.000012000
6       -0.650927000     -1.201237000     -0.000012000
6       -1.378444000     -0.019084000      0.000001000
6        0.603937000      1.324348000     -0.000013000
6       -0.792009000      1.245060000     -0.000001000
7        1.553362000     -2.191367000      0.000081000
1        1.066412000      2.299037000     -0.000025000
1       -2.459976000     -0.082442000      0.000016000
6       -1.451144000     -2.514618000     -0.000008000
6       -2.342560000     -2.543733000     -1.257816000
6       -2.342280000     -2.543868000      1.257991000
1       -1.729155000     -2.514181000      2.161939000
1       -3.039134000     -1.704429000      1.292372000
1       -2.927254000     -3.467397000      1.271006000
1       -3.039429000     -1.704301000     -1.291970000
1       -2.927536000     -3.467261000     -1.270793000
1       -1.729633000     -2.513960000     -2.161895000
6        2.942339000      0.326292000     -0.000010000
6        3.527814000     -0.335299000     -1.262062000
6        3.527828000     -0.335244000      1.262066000
1        3.110330000      0.122279000      2.163123000
1        3.320445000     -1.404777000      1.285550000
1        4.612063000     -0.192604000      1.278213000
1        3.320418000     -1.404831000     -1.285506000
1        4.612051000     -0.192675000     -1.278221000
1        3.110319000      0.122195000     -2.163135000
6       -0.621527000     -3.804163000     -0.000188000
1        0.015067000     -3.882788000      0.881623000
1       -1.313239000     -4.652127000     -0.000247000
1        0.014972000     -3.882589000     -0.882087000
6        3.393791000      1.793532000     -0.000044000
1        3.047887000      2.327607000     -0.888848000
1        3.047909000      2.327639000      0.888750000
1        4.485435000      1.824477000     -0.000058000
6       -1.686854000      2.485175000      0.000003000
6       -2.575410000      2.467469000      1.256457000
6       -2.575437000      2.467459000     -1.256432000
1       -1.963614000      2.472446000     -2.161806000
1       -3.216491000      1.584095000     -1.286405000
1       -3.219017000      3.351410000     -1.270131000
1       -3.216463000      1.584105000      1.286451000
1       -3.218991000      3.351420000      1.270163000
1       -1.963568000      2.472464000      2.161818000
6       -0.880963000      3.788120000     -0.000011000
1       -0.248629000      3.871525000      0.887514000
1       -0.248648000      3.871516000     -0.887550000
1       -1.568130000      4.637395000     -0.000008000


48
```

TS1d, i568.9 cm-1, (UM062X) = -757.87103

6	-0.668594000	1.216658000	0.143459000
6	-1.195584000	-0.110869000	0.491438000
6	-0.504235000	-1.329824000	0.039365000
6	0.823842000	-1.156521000	-0.144017000
6	0.750233000	1.267419000	0.186580000
6	1.495887000	0.117860000	0.057999000
7	-1.905598000	0.194094000	1.479582000
1	1.430429000	-2.006444000	-0.433247000
1	1.244101000	2.231060000	0.214246000
6	-1.509974000	2.441705000	-0.170795000
6	-0.894494000	3.121620000	-1.411902000
6	-1.489670000	3.431201000	1.005211000
1	-1.954985000	2.976153000	1.881080000
1	-0.466492000	3.716068000	1.265305000
1	-2.037971000	4.340305000	0.740934000
1	0.120858000	3.476189000	-1.226027000
1	-1.503436000	3.985637000	-1.689395000
1	-0.866328000	2.433070000	-2.260224000
6	-1.291829000	-2.609337000	-0.132480000
6	-2.428605000	-2.351811000	-1.137709000
6	-1.898174000	-2.992908000	1.228699000
1	-1.110335000	-3.200463000	1.956522000
1	-2.523823000	-2.191665000	1.628547000
1	-2.514709000	-3.889217000	1.116500000
1	-3.090646000	-1.557775000	-0.783505000
1	-3.023477000	-3.260001000	-1.266256000
1	-2.028505000	-2.059902000	-2.112198000
6	-2.960602000	2.076146000	-0.505636000
1	-3.483631000	1.683724000	0.364775000
1	-3.483115000	2.970288000	-0.855017000
1	-2.999584000	1.326458000	-1.301500000
6	-0.415544000	-3.753014000	-0.643981000
1	0.030992000	-3.510921000	-1.612203000
1	0.385844000	-3.988131000	0.061259000
1	-1.024310000	-4.651793000	-0.768225000
6	3.021231000	0.120966000	0.029126000
6	3.605245000	1.514201000	0.280239000
6	3.512765000	-0.365916000	-1.346637000
1	3.190138000	-1.386565000	-1.560565000
1	3.137584000	0.283390000	-2.141510000
1	4.605716000	-0.349162000	-1.375293000
1	3.324462000	2.217542000	-0.507687000
1	4.695831000	1.451897000	0.295191000
1	3.278110000	1.917970000	1.241527000
6	3.542694000	-0.826414000	1.125200000
1	3.211416000	-0.492580000	2.111423000
1	3.191491000	-1.849885000	0.979095000
1	4.636007000	-0.840887000	1.114420000

48

2d, E(RM062X) = -757.90164

6	0.471819000	1.367308000	-0.458684000
6	1.109896000	0.064475000	-0.669842000
6	0.754297000	-1.219972000	-0.116148000
6	-0.582923000	-1.232925000	0.103493000
6	-0.980543000	1.160607000	-0.341950000
6	-1.476492000	-0.085743000	-0.115426000
7	1.147824000	0.769082000	-1.708578000
1	-1.033951000	-2.153637000	0.455401000
1	-1.643364000	2.018618000	-0.336002000
6	1.130519000	2.567600000	0.215576000
6	2.654309000	2.454900000	0.141015000
6	0.698732000	2.612239000	1.687552000
1	-0.384194000	2.725098000	1.782560000
1	0.987003000	1.689608000	2.200343000
1	1.172916000	3.454591000	2.199203000
1	3.008111000	1.559885000	0.661848000
1	3.111560000	3.326471000	0.615928000
1	2.997426000	2.402775000	-0.894119000
6	1.765239000	-2.318634000	0.112451000
6	2.854716000	-1.785154000	1.057235000
6	2.399653000	-2.673126000	-1.242470000
1	1.649998000	-3.076941000	-1.926643000
1	2.844696000	-1.790570000	-1.709553000
1	3.185205000	-3.421265000	-1.105371000
1	3.351730000	-0.913435000	0.622736000

1	3.611693000	-2.554750000	1.230096000
1	2.427985000	-1.494746000	2.020605000
6	0.686145000	3.848823000	-0.503331000
1	-0.397552000	3.982710000	-0.450858000
1	1.151781000	4.725654000	-0.044598000
1	0.975666000	3.813201000	-1.556352000
6	1.118064000	-3.559488000	0.725704000
1	0.354849000	-3.978081000	0.064723000
1	1.878119000	-4.327536000	0.887609000
1	0.656850000	-3.331118000	1.690161000
6	-2.976204000	-0.365958000	0.019622000
6	-3.823692000	0.883725000	-0.235881000
6	-3.288286000	-0.870145000	1.439790000
1	-2.775268000	-1.806277000	1.669026000
1	-2.988849000	-0.128258000	2.184492000
1	-4.362211000	-1.048191000	1.543638000
1	-3.633600000	1.659586000	0.509991000
1	-4.882694000	0.620989000	-0.177819000
1	-3.632940000	1.299793000	-1.228068000
6	-3.381547000	-1.436278000	-1.008865000
1	-3.175245000	-1.088737000	-2.024102000
1	-2.841646000	-2.373514000	-0.857245000
1	-4.451068000	-1.649018000	-0.926650000

B. CBS-QB3 Geometries

The CBS-QB3 method uses geometries optimized with the B3LYP functional and the 6-311G(2d,d,p) basis set. For hydrogen and first row atoms this basis set equates to 6-311G(d,p).

```
12
Triplet 1a, E(UB3LYP) =  -286.37717
6        0.000000000      1.230868000      0.336085000
6        0.000000000      0.000000000      1.071696000
6        0.000000000     -1.230868000      0.336085000
6        0.000000000     -1.214439000     -1.044317000
6        0.000000000      1.214439000     -1.044317000
6        0.000000000      0.000000000     -1.743925000
1        0.000000000      0.000000000     -2.827410000
7        0.000000000      0.000000000      2.394435000
1        0.000000000      2.150470000     -1.591475000
1        0.000000000      2.160765000      0.890732000
1        0.000000000     -2.160765000      0.890732000
1        0.000000000     -2.150470000     -1.591475000
```

```
12
TS1a, i662.5 cm-1, E(UB3LYP) =  -286.33108
6        0.682395000     -1.007848000     -0.394376000
6        1.023277000      0.396061000     -0.145836000
6       -0.001182000      1.426180000     -0.207147000
6       -1.267070000      0.980184000      0.006572000
6       -0.615672000     -1.391270000      0.022401000
6       -1.561709000     -0.412536000      0.230473000
1       -2.579060000     -0.689468000      0.480384000
7        2.053080000      0.100809000      0.515716000
1        0.241383000      2.466887000     -0.379672000
1       -2.088940000      1.687749000      0.022324000
1       -0.897720000     -2.438966000      0.043306000
1        1.392535000     -1.676499000     -0.848877000
```

```
12
2a, E(RB3LYP) =  -286.34636
6       -1.119268000      0.576973000     -0.413768000
6       -0.746395000     -0.834081000     -0.113381000
6        0.556277000     -1.416771000     -0.214958000
6        1.512132000     -0.478235000      0.034771000
6       -0.021826000      1.460663000     -0.058377000
6        1.203796000      0.924420000      0.222892000
1        2.027068000      1.583724000      0.473949000
7       -1.670151000     -0.446572000      0.642230000
1        0.756713000     -2.453689000     -0.446339000
1        2.553809000     -0.778981000      0.072744000
1       -0.131418000      2.537980000     -0.144227000
1       -1.823409000      0.839165000     -1.194808000
```

```
12
TS2a, i477.2 cm-1, E(RB3LYP) =  -286.34033
6        1.283237000      0.508914000      0.331278000
6        0.564291000     -1.049419000      0.036642000
6       -0.755433000     -1.327615000      0.296997000
6       -1.560763000     -0.244133000     -0.076457000
6        0.245822000      1.415565000      0.125474000
6       -1.053799000      1.050964000     -0.260949000
1       -1.760013000      1.840171000     -0.490742000
7        1.526500000     -0.634715000     -0.622679000
1        0.405861000      2.437705000      0.460585000
1        2.073529000      0.671361000      1.055348000
1       -1.111495000     -2.236160000      0.759843000
1       -2.633509000     -0.395735000     -0.144192000
```

```
12
3a, E(RB3LYP) =  -286.35751
6        1.589447000      0.122999000      0.146304000
```

6	-0.012849000	-1.309439000	-0.034215000
6	-1.236966000	-1.002504000	0.348125000
6	-1.569223000	0.343063000	-0.148643000
6	0.777951000	1.203989000	0.233538000
6	-0.621828000	1.314885000	-0.175482000
1	-0.927337000	2.303784000	-0.506577000
7	1.131337000	-1.086639000	-0.461762000
1	-2.576699000	0.565619000	-0.492289000
1	-1.855630000	-1.583577000	1.016494000
1	1.246923000	2.126944000	0.562127000
1	2.634193000	0.155747000	0.434821000

12
3a, Triplet state, E(UB3LYP) = -286.32775

6	-1.253374000	1.004953000	0.000000000
6	-0.823549000	-1.333650000	0.000000000
6	0.571404000	-1.555421000	0.000000000
6	1.507581000	-0.504909000	0.000000000
6	0.000000000	1.533138000	0.000000000
6	1.271090000	0.858936000	0.000000000
1	2.143013000	1.503245000	0.000000000
7	-1.601256000	-0.347903000	0.000000000
1	2.553874000	-0.804345000	0.000000000
1	0.932168000	-2.576360000	0.000000000
1	0.046105000	2.618653000	0.000000000
1	-2.105277000	1.675845000	0.000000000
